# Subjective Socioeconomic Status Moderates How Resting Heart Rate Variability Predicts Pain Response

**DOI:** 10.1007/s42761-023-00234-w

**Published:** 2024-01-19

**Authors:** Jacinth J. X. Tan, Chin Hong Tan, Michael W. Kraus

**Affiliations:** 1 School of Social Sciences, Singapore Management University, 10 Canning Rise, #05-01, Singapore, 179873 Singapore; 2 Department of Psychology, Nanyang Technological University, Singapore, Singapore; 3 Lee Kong Chian School of Medicine, Nanyang Technological University, Singapore, Singapore; 4 School of Management, Yale University, New Haven, CT USA; 5 Department of Psychology, Northwestern University, Evanston, IL USA

**Keywords:** Socioeconomic status, Pain, Stress, Heart rate variability

## Abstract

Higher resting heart rate variability (HRV)—an index of more flexible response to environmental stressors, including noxious stimuli—has been linked to reduced perception of experimentally induced pain. However, as stress responses are adapted to one’s chronic environments, we propose that chronic exposure to threats captured by one’s subjective socioeconomic status (SSS) may shape different adaptations that produce distinct pain responses linked to higher resting HRV. Specifically, lower SSS individuals with more threat exposures may prioritize threat detection by upregulating sensitivity to stressors, such as acute pain. Therefore, higher HRV would predict greater perceived acute pain among lower SSS individuals. In contrast, higher SSS individuals with less threat exposures may instead prioritize affective regulation by downregulating sensitivity to stressors, producing lower pain perception with higher HRV. We examined this stress response moderation by SSS in 164 healthy young adults exposed to experimental pain via the cold pressor test (CPT). Resting HRV, indexed by the *root-mean-square of successive differences* in heart rate, and self-reported SSS were measured at rest. Pain perception indexed by self-reported pain and pain tolerance indexed by hand-immersion time during the CPT were assessed. Results revealed that among higher SSS individuals, higher resting HRV predicted lower pain reports and subsequently greater pain tolerance during the CPT. Conversely, among lower SSS individuals, higher resting HRV predicted higher pain reports and subsequently lower pain tolerance. These findings provide preliminary evidence that environmental stress exposures linked to one’s SSS may shape unique biological adaptations that predict distinct pain responses.

**Supplementary Information:**

The online version contains supplementary material available at 10.1007/s42761-023-00234-w.

Human biological systems evolve to respond to environmental demands and stressors in adaptive ways (Ellis et al., 2017). In particular, parasympathetic nervous system (PNS) activity that modulates control of the cardiovascular system—among multiple other organs and bodily systems—by the vagus nerve (Berntson et al., 1997) has been linked to better affective regulation and recovery in response to stress (Balzarotti et al., 2017; Gottman & Katz, 2002). While the sympathetic nervous system (SNS) activity innervates the heart to increase heart rate, PNS activity independently decreases heart rate via inhibitory control of the heart by the vagus nerve (Weissman & Mendes, 2021). Generally, higher PNS activity at rest allows the body to stay calm and conserve energy, such that vagal control can be rapidly and flexibly engaged to influence heart rate in response to dynamic changes to one’s environment (Grossman & Taylor, 2007; Thayer & Lane, 2000). Therefore, higher resting PNS is often taken to indicate a highly adaptable biological system to environmental demands and stressors.

The adaptive capacity of the PNS is often assessed by a person’s heart rate variability (HRV)—changes between successive heartbeats—at rest, with higher HRV indicating higher adaptive ability. For instance, higher resting HRV has been linked to enhanced ability to detect and discriminate socio-emotional cues (Beauchaine, 2001; Park et al., 2012), and more successful emotion regulation (Mather & Thayer, 2018). In terms of general stress response, higher resting HRV has been linked to dampened stress responses, including to noxious lab stimulus. For instance, studies have shown that in healthy individuals, higher HRV predicted lower sensitivity to experimentally induced acute pain (Appelhans & Luecken, 2008; Koenig et al., 2014). The proposed explanation in these studies is that higher HRV reflects the engagement of affective regulation processes that modulate pain perception (Appelhans & Luecken, 2006; Mather & Thayer, 2018). Suggestive of this, one neuroimaging study of healthy participants found that in response to an experimentally induced visceral pain, higher HRV was linked to stronger connectivity in brain regions involved in engaging affective or arousal modulation (e.g., the thalamus-amygdala, thalamus-hypothalamus, hypothalamus-nucleus accumbens; Ruffle et al., 2018).

In the above studies involving pain perception, adaptive responses linked to higher resting HRV involve affective regulation, specifically via *downregulation*, which produces dampened perception of pain. However, according to the adaptation-based approach to resilience (Ellis et al., 2017), frequent stress exposures over time can shape heightened sensitization to the environment to facilitate stress response—a useful adaptation for individuals in chronically stressed environments. Therefore, one unexamined possibility is that under conditions where sensitivity to one’s environment is important—such as high exposure to environmental threat—higher HRV may involve engaging in affective *upregulation*, to heighten perception and sensitivity necessary for threat detection.

A person’s chronic environmental exposure can differ by their subjective socioeconomic status (SSS), which encompasses one’s perceived availability of material resources and overall perceived societal rank relative to others (Cundiff & Matthews, 2017; Tan et al., 2020). Lower SSS individuals, who perceive having fewer material resources and lower societal rank, tend to experience more negative affect, particularly feelings and expectations of threat, compared to higher SSS individuals (Adler & Snibbe, 2003; Operario et al., 2004). Over time, such chronic threat experiences have been argued to shape general threat vigilance—an orientation toward rapid threat detection and response facilitation—among lower SSS individuals (Kraus et al., 2011). Consistent with this view, lower SSS individuals have been found to be more sensitive to their social environments, such as being more attentive to people on city streets (Dietze & Knowles, 2016) and track the negative emotions of others more accurately (Kraus et al., 2011) than higher SSS individuals.

Given that biological systems adapt in response to chronic environmental exposures (Ellis et al., 2017) and these exposures differ by SSS, we propose that adaptation patterns linked to higher resting HRV that shape stress response, such as pain perception, may differ by one’s SSS. Specifically, for lower SSS individuals who contend with more environmental threats (Adler & Snibbe, 2003; Kraus et al., 2011), higher HRV affords the ability to efficiently mobilize energy in response to environmental demands—specifically for rapid threat detection and dealing with the threat to minimize its impact. Therefore, for lower SSS individuals, higher HRV should predict affective *upregulation* via heightened perception of a noxious stimulus, as well as rapid disengagement from it. Conversely, for higher SSS individuals who contend with less environmental threats, higher HRV may be adapted for mobilizing energy for stress modulation (Appelhans & Luecken, 2006; Mather & Thayer, 2018). Therefore, higher HRV should predict affective downregulation via reduced perception of a noxious stimulus, and greater ability to withstand the stimulus, among higher SSS individuals.

In this research, we sought to test our theory of distinct SSS adaptations to stress by examining the SSS moderation hypothesis—whether resting HRV predicts different patterns of pain response as a function of a person’s SSS. To this end, we exposed participants to experimentally induced pain—a noxious lab stimulus—via the cold pressor test (CPT), to study their pain response. The CPT involves immersing one’s hand in an ice water bath maintained at a temperature range over a period of time (Wirch et al., 2006). It reliably elicits acute pain and autonomic stress (Silverthorn & Michael, 2013). We examined two key pain responses: *pain perception* assessed by participants’ subjective pain reports and *pain tolerance* as a behavioral measure of stimulus disengagement, indexed by how long participants kept their hand immersed in the ice water bath. Participants’ resting heart rate was recorded continuously for 5 min using an electrocardiogram (ECG) before the CPT. Resting HRV was derived from resting heart rate using the *root-mean-square of successive differences* (RMSSD) index—the changes in intervals between successive heartbeats—a time domain measure of vagally mediated HRV (Koenig et al., 2014). Participants reported their SSS at the end of the study. We hypothesized that the relationship between resting HRV and pain responses will be moderated by participants’ SSS. Specifically, higher resting HRV will predict higher pain reports and lower pain tolerance (i.e., more rapid disengagement) for lower SSS individuals, but lower pain reports and higher pain tolerance for higher SSS individuals.

## Method

### Participants

One hundred and sixty-nine young adults were recruited from a campus town community (full sample characteristics in Table 1). We did not determine the sample size a priori and recruited as many participants as we could across two semesters. We focused on healthy and pain-free young adults whose stress responses are less influenced by their health status. Five participants’ data were excluded due to excessive irregularities in their physiological signals. The final sample size was 164. Participants were paid US $10. The study was approved by the University of Illinois, Urbana Champaign (UIUC) Institutional Review Board.

**Table 1:** Sample characteristics

Characteristics	Levels	Percentage	*M*	*SD*
Sex	Female	65.1		
	Male	34.9		
Age			20.69	3.74
Race	African American	10.2		
	Asian American	19.3		
	European American	51.2		
	Latino/a	10.8		
	Other	8.4		
Subjective socioeconomic status (SSS)			6.31	1.51
Resting HRV			44.09	19.54

We determined the smallest effect size that can be detected with the current sample size of 164 via a sensitivity analysis using G*Power (Faul et al., 2009). Assuming a maximum of eight predictors (two main effects, one interaction effect, and up to four covariates) in a linear regression, using a two-tailed test at 80% power and alpha-level at .05, the smallest effect size that can be detected in this study is *R*^2^ = .046 (*f*^2^ = .048).

### Procedure

Participants were run individually in the psychology lab. Participants first provided their informed consent and then answered health screening questions in a private room. Those who reported not currently taking any medication and no current/history of physical injuries/cardiovascular conditions continued with the study. After successful screening, physiological sensors were applied to participants using a Lead II configuration, which were attached to an electrocardiogram (ECG) to track their continuous heart rate. ECG signals were recorded at 1,000 Hz with the Biopac MP160 hardware (Biopac, Inc., Goleta, CA). After signal checks, participants sat alone for a 5-min baseline recording. After baseline, participants were brought to the next room, where an ice water bath maintained at 0 to 2 °C—which is within the recommended range (Wirch et al., 2006)—was set up to perform the CPT. Participants were first asked to rate their current pain level on a pain scale. Then, they were instructed to immerse their hand fully in the ice water bath for as long as they can. Once participants’ hands reached the base of the water bath, the experimenter started the timer. Once their hand was removed, the timer was stopped. Immediately, participants were asked to rate their current pain level on the same pain scale. Participants returned to the first room for a 5-min recovery and then answered survey questions about their personality and demographic information, including their SSS. Finally, participants were paid and debriefed.

### Measures

#### Resting HRV

Resting HRV was assessed by RMSSD derived from the 5-min ECG recording at baseline (Berntson et al., 1997). All ECG waveforms were visually inspected offline for correct identification of ECG R-peaks. Misidentified R-peaks were edited. Inspected ECG waveforms were then scored in 60-s bins, generating five RMSSD bin scores. All signal editing and scoring were done using the software Acqknowledge 5 (Biopac, Inc., Goleta, CA). The five RMSSD bin scores were averaged to index resting HRV (*M* = 44.09, *SD* = 19.54). Five participants had excessively noisy bins that could not be reliably edited and were dropped from the analyses. The RMSSD scores were slightly skewed and were log-transformed to reduce skew (Siennicka et al., 2019; Sin et al., 2016; Thorson et al., 2020; *M* = 3.68, *SD* = 0.49). We report analyses and results using the raw scores here, and those using transformed scores in the Supplemental Online Material.

#### Pain Reports

Perceived pain was measured using the Faces Pain Rating scale (Wong & Baker, 1988). This measure combines pictures of faces that range from a smiling face to a crying face, with a number that corresponds to each face (0 = *no hurt/smiling face*; 10 = *hurts worst/crying face*). Participants reported their pain level twice: right before (time 1 *M* = 0.09, *SD* = 0.47) and right after (time 2 *M* = 5.53, *SD* = 1.97) the CPT.

#### Pain Tolerance

Pain tolerance was assessed as their immersion time (in seconds) in the ice water bath (*M* = 77.25; *SD* = 69.89). Longer immersion time indicated higher pain tolerance.

#### Subjective Socioeconomic Status (SSS)

SSS was measured using the MacArthur Scale of Subjective Status (Adler et al., 2000), which presents a ten-rung ladder described as representing different social ranks in the USA. Those at the highest rung (10 = *very top*) have the highest incomes, highest educational attainment, and the best jobs, while those at the lowest rung (1 = *very bottom*) have the lowest incomes, lowest educational attainment, and the worst jobs. Participants rated where they stood on this ladder (*M* = 6.31; *SD* = 1.51).

#### Covariates

Age and sex were assessed for inclusion as covariates in the analyses, given their possible links to both SSS (Operario et al., 2004) and pain perception (Lautenbacher et al., 2017; Racine et al., 2012).

### Analytic Strategy

Multiple regression analyses were conducted on pain reports and pain tolerance as dependent variables. Prior to analysis, SSS and resting HRV were mean-centered. Two models were estimated: Model 1 included age and sex as covariates, and SSS and HRV as predictors, which tested the independent effects of SSS and HRV. Model 2 included an SSS by resting HRV interaction as a predictor, to evaluate our hypothesis. When the interaction effect was significant, simple slopes were analyzed to determine how resting HRV predicted the dependent variables at higher SSS (mean + 1 *SD*) and at lower SSS (mean − 1 *SD*; Aiken & West, 1991).

## Results

Table 2 presents the zero-order correlations between all variables of interest.

**Table 2:** Zero-order correlations between all study variables

	Resting HRV	Pain report	Immersion time	SSS
Resting HRV	1			
Pain report	− .10	1		
Immersion time	.014	− .34**	1	
SSS	.026	− .058	.052	1

*SES* socioeconomic status

**p < .001

### Results for Perceived Pain Reports

**Fig. 1:**
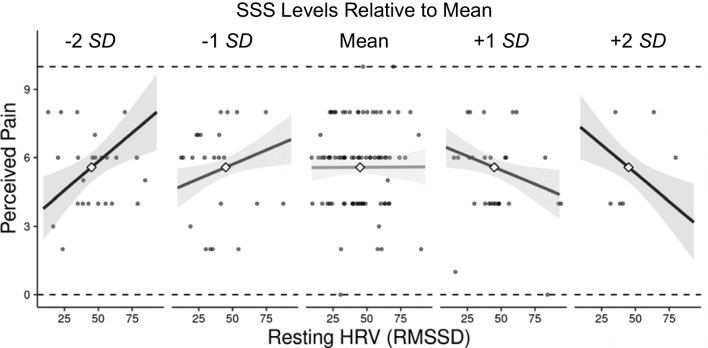
The relationship between resting HRV and perceived pain as a function of SSS levels. Plots were generated using https://connorjmccabe.shinyapps.io/interactive/

In Model 1, resting HRV, SSS, and all covariates did not significantly predict pain reports, *p*s > .19. Critically, in Model 2, there was a significant interaction effect between resting HRV and SSS, *β* =  − 0.017, *t* (158) =  − 3.33, *p* = .001, 95% CI [− 0.026, − 0.010], *R*^2^_adjusted_ = .080. The size of this effect is larger than the minimum effect size that can be detected by the current sample size estimated by our power analysis. Consistent with our hypothesis, simple slope analysis revealed that at higher SSS, higher resting HRV predicted significantly lower pain perceived, *β* =  − 0.024, *t* (158) =  − 2.29, *p* = .023, 95% CI [− 0.045, − 0.0033]. At lower SSS, an opposite pattern emerged, such that higher resting HRV predicted significantly higher pain perceived, *β* = 0.026, *t* (158) = 2.40, *p* = .018, 95% CI [0.046, 0.47] (see Fig. 1). The full model parameters are presented in Table 3.

**Table 3:** Model summaries of perceived pain with SSS and resting HRV as predictors

	Model 1				Model 2			
Predictors	*B*	*SE*	*t*	*p*	*B*	*SE*	*t*	*p*
Controls								
Age	0.039	0.040	0.97	.34	0.052	0.039	1.31	.19
Gender	0.36	0.32	1.11	.27	.31	0.31	1.01	0.31
Main effects								
SSS	− 0.040	0.10	− 0.40	.69	0.014	0.10	0.14	.89
Resting HRV	− 0.001	0.008	0.053	.96	0.001	0.008	0.10	.92
Interaction								
SSS × resting HRV					− **0.017**	0.005	− 3.33	.001

Estimates in bold are statistically significant

In our proposed theory, we argued that subordinate rank captured by SSS plays a more central role in eliciting chronic perceptions of threat, which shapes sensitivity and affects upregulation. This is consistent with the psychological perspective of class captured by subjective measures, which has been theorized to capture feelings of subordinate rank relative to others—distinct from objective resources (e.g., Tan et al., 2020). Nonetheless, we conducted parallel analyses with objective measures of socioeconomic status collected as part of the demographic questions—specifically reported annual household income and parent’s highest educational attainment. Perceived pain was not significantly predicted by any of the objective measures directly or interactively with resting HRV. This may be unsurprising as the SSS and the objective measures were not strongly correlated (*r* = .24 with household income and *r* = .39 with parents’ education level). To demonstrate the central and unique role of SSS, we conducted a parallel analysis where we additionally controlled for objective measures of reported income and parent’s educational attainment. The key hypothesized patterns with SSS and pain perception moderated by HRV held. The full details of these additional analyses involving objective socioeconomic status measures are reported in the Supplemental Online Material.

### Results for Pain Tolerance

There were no significant effects of SSS and resting HRV (including covariates) on immersion time, across all models (all *p*s > .81). In other words, SSS and HRV did not directly predict pain tolerance. To assess whether SSS and HRV might predict pain tolerance *indirectly* through reported pain, we conducted a moderated path analysis (Edwards & Lambert, 2007). We estimated the indirect pathways from resting HRV to immersion time via reported pain levels at higher SSS (mean + 1 *SD*) and at lower SSS (mean − 1 *SD*), controlling for age and sex. Confidence intervals of indirect path estimates were generated via bootstrapping to test if each indirect path was significant, and if both paths differed significantly from each other.

**Fig. 2:**
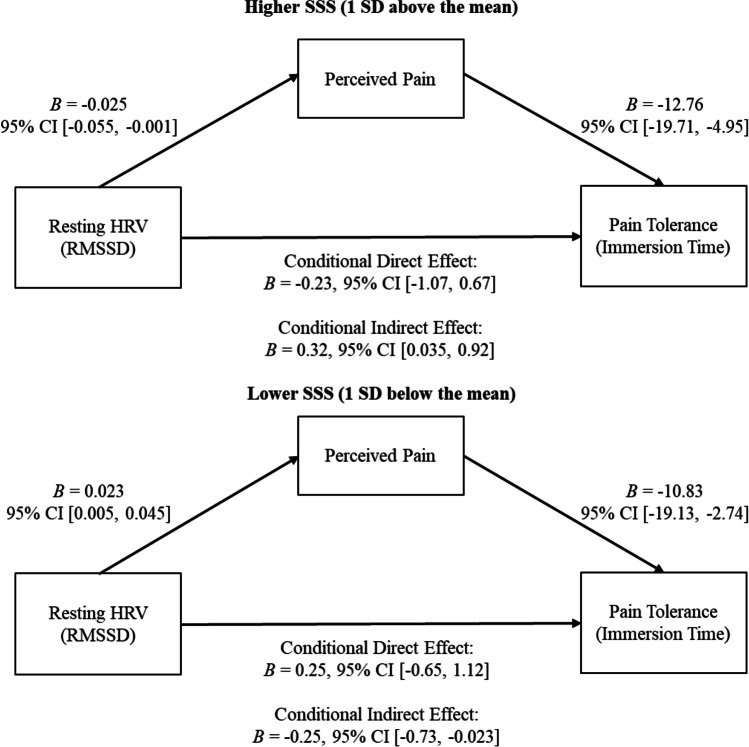
The indirect effect of resting HRV on pain tolerance through pain reports at high SSS and at low SSS

This analysis revealed a significant indirect effect of resting HRV at higher SSS, *B* = 0.32, 95% CI [0.035, 0.92]: Higher resting HRV predicted lower pain reports, *B* =  − 0.025, 95% CI [− 0.055, -0.001], and subsequently longer immersion time (i.e., higher pain tolerance), *B* =  − 12.76, 95% CI [− 19.71, − 4.95]. At lower SSS, the indirect effect of resting HRV was significant but opposite, *B* =  − 0.25, 95% CI [− 0.73, − 0.023]: Higher resting HRV predicted higher pain reports, *B* = 0.023, 95% CI [0.005, 0.045], followed by shorter immersion time (i.e., lower pain tolerance), *B* =  − 10.83, 95% CI [− 19.13, − 2.74]. The overall index of moderated pathway by SSS was significant, *B* = 0.57, 95% CI [0.20, 1.34], indicating that both pathways were different from each other (see Fig. 2).

## Discussion

This research examined how pain perception may be predicted by distinct stress adaptations linked to higher resting HRV shaped by one’s SSS. Consistent with our theory that stress responses are uniquely adapted to one’s SSS exposures, we observed that higher resting HRV was linked to threat detection and minimization for lower SSS individuals via affective upregulation, which predicted heightened pain perception and subsequently lower pain tolerance (i.e., rapid disengagement). Conversely, higher resting HRV was linked to affective downregulation via reduced pain perception and subsequently higher pain tolerance for higher SSS individuals. Overall, our sample provided sufficient power to detect the effects we observed. In contrast to past studies that observed downregulated responses linked to higher HRV when participant backgrounds are aggregated—assuming similar environmental exposures and adaptations—we provide novel evidence that biological adaptations may produce upregulated or downregulated responses depending on individuals’ environmental stress exposures, indexed by SSS.

Although pain is widely recognized as a psychosomatic experience, only in recent years did the understanding of pain shift from one that is purely biological or psychosocial in nature to one that considers their interactive role (Raja et al., 2020; Williams & Craig, 2016). In this expanded view, pain has both physiological underpinnings, such as in nociceptive pain that involves tissue damage due to an injury or medical condition, as well as psychosocial underpinnings, as a result of psychological responses linked to one’s life experiences, such as chronic stress and hardship that increase pain tolerance (Raja et al., 2020), or adaptive psychological responses that modulate arousal and therefore pain responses. This suggests that both physiological and psychological factors must be considered to gain a comprehensive understanding of pain experiences. The current findings from this research provide evidence for this perspective, that pain responses can be better characterized and distinguished when the biological role of resting HRV and the psychosocial role of SSS are jointly considered.

Not all lower SSS individuals show sensitization to stress, and some may exhibit desensitization. This may be understood within the adaptive calibration model (ACM; Del Guidice et al., 2011)—an evolutionary-based model that proposes two patterns of stress adaptation to early adversity: the *vigilance* response, characterized by heightened sensitivity to stress, and the *unemotional* response, characterized by dampened sensitivity to stress. These responses are theorized to follow a developmental trajectory, with vigilance developing in early childhood, and then a possible shift to unemotional later especially under long-term stress. Our current theory and findings with lower SSS young adults are consistent with the vigilance adaptation of the ACM. However, if stress exposures endure in later life—due to the inability to remove oneself from or deal with threats—lower SSS individuals may transition to a desensitized, unemotional response. Within the ACM, such desensitization stems from inhibiting or blocking information about environmental threats, which is expected to predict maladaptive behaviors, such as greater risk-taking and antisocial behaviors (Ellis & Del Giudice, 2019). This theoretical extension could ideally be examined by tracking the life experiences and behaviors of lower SSS individuals longitudinally from early childhood to midlife.

The lack of significant associations between resting HRV and pain responses in our study unlike in past studies (e.g., Appelhans & Luecken, 2008; Koenig et al., 2014) was unexpected. This may be due to our relatively young and healthy adult sample having a more limited HRV range, and in such populations, one’s SSS matters in understanding how pain perception may differ by HRV. The lack of basic SSS differences in pain reports and tolerance may also be surprising, given past documentation of pain and stress disparities by social class (Adler & Snibbe, 2003; Beshai et al., 2017; Caner & Yiğit, 2019). However, we note that these past studies examined *frequency* of pain experiences, which often varied by SES due to differences in environments and negative exposures. In contrast, our study examined SSS differences in pain *perception* of the *same* negative exposure—which has not been examined to our knowledge. Therefore, our finding that pain perception differed not simply by SSS but by an interaction between SSS and resting HRV demonstrates a novel phenomenon.

There are some limitations of this research. First, the research is correlational in nature and cannot clearly demonstrate that pain perception is causally shaped by HRV and SSS. Examining these relationships longitudinally, especially from an early age, would be a complementary approach to better ascertain how HRV may be adapted to SSS over time to shape affective regulation and pain perception. Second, in addition to our restricted age range, our research also comprised largely of White participants. Finally, as this is the first novel demonstration of how SSS interacts with resting HRV to predict pain responses, it is important to provide more converging support by directly replicating these findings with a larger, as well as more age, and ethnically diverse sample.

Despite the limitations, the current findings offer a preliminary novel insight into how pain perception is linked to individual differences in biological adaptations to stress. We believe future research that adopts a similar individual difference approach will extend our understanding of how individual and psychosocial factors may uniquely shape how our biological systems adapt and respond to stress.

### Supplementary Information

Below is the link to the electronic supplementary material.

Supplementary file1 (DOCX 94 KB)

## Additional Information

### Availability of data and material

All individual de-identified participant data, analysis code, and research materials reported in this study are shared at https://osf.io/d7n56/.
