# Correction To: Subjective Socioeconomic Status Moderates How Resting Heart Rate Variability Predicts Pain Response

**DOI:** 10.1007/s42761-024-00236-2

**Published:** 2024-02-12

**Authors:** Jacinth J. X. Tan, Chin Hong Tan, Michael W. Kraus

**Affiliations:** 1 School of Social Sciences, Singapore Management University, 10 Canning Rise, #05-01, Singapore, 179873 Singapore; 2 Department of Psychology, Nanyang Technological University, Singapore, Singapore; 3 Lee Kong Chian School of Medicine, Nanyang Technological University, Singapore, Singapore; 4 School of Management, Yale University, New Haven, CT USA; 5 Department of Psychology, Northwestern University, Evanston, IL USA

**Correction To: Affective Science**

10.1007/s42761-023-00234-w

The article “Subjective Socioeconomic Status Moderates How Resting Heart Rate Variability Predicts Pain Response,” written by IJacinth J. X. Tan, Chin Hong Tan, and Michael W. Kraus, was originally published electronically on the publisher’s internet portal on 19 January 2024 without open access. With the author(s)’ decision to opt for Open Choice, the copyright of the article changed on 22 January 2024 to © The Author(s) 2024, and the article is forthwith distributed under a Creative Commons Attribution 4.0 International License, which permits use, sharing, adaptation, distribution, and reproduction in any medium or format, as long as you give appropriate credit to the original author(s) and the source, provide a link to the Creative Commons license, and indicate if changes were made. The images or other third-party material in this article is included in the article’s Creative Commons license, unless indicated otherwise in a credit line to the material. If material is not included in the article’s Creative Commons license and your intended use is not permitted by statutory regulation or exceeds the permitted use, you will need to obtain permission directly from the copyright holder. To view a copy of this license, visit http://creativecommons.org/licenses/by/4.0. Open access funding enabled and organized by Projekt DEAL.

The original article has been corrected.

