# Supplementary material for: Subjective Socioeconomic Status Moderates How Resting Heart Rate Variability Predicts Pain Response
Source: Affect Sci. 2024 Jan 19;5(2):1–8. doi: 10.1007/s42761-023-00234-w (PMC11264642; doi:10.1007/s42761-023-00234-w)
Supplement: Supplementary file 1 — Supplementary file1 (DOCX 94 KB) [file 42761_2023_234_MOESM1_ESM.docx]

**Supplemental Online Material**

**Analysis using log-transformed RMSSD as HRV.** As reported in the main text, the RMSSD scores were slightly skewed and were log-transformed to reduce skew (Siennicka et al., 2019; Sin et al., 2016; Thorson et al., 2019; *M* = 3.68, *SD* = 0.49). We conducted a parallel analysis for perceived pain and pain tolerance with this log-transformed variable.

For perceived pain, there were no significant effects of SSS, log-transformed RMSSD as HRV, and the covariates in Model 1. In Model 2, there was a significant interaction effect between HRV and SSS, β = -0.65, *t*(158) = -3.09, *p* = .002, 95% CI [-1.07, -.23], *R*^2^_adjusted_ = .072. Similar to the analysis using raw RMSSD as HRV reported in the main text, higher resting HRV predicted significantly lower pain perceived at higher SSS, β = -0.93, *t*(158) = -2.06, *p* = .041, 95% CI [-1.82, -.037]. The opposite pattern emerged at lower SSS, such that higher resting HRV predicted significantly higher pain perceived, β = 1.04, *t*(158) = 2.43, *p* = .016, 95% CI [.19, 1.89]. The full model parameters are presented in Table S1. In addition, Figure S1 provides a plot of regions of significance of the HRV simple slopes at low and high SSS.

| Table S1. Model Summaries of Perceived Pain with SSS and Log-Transformed RMSSD as HRV as Predictors. | | | | | | | | | | | | | | | |
| --- | --- | --- | --- | --- | --- | --- | --- | --- | --- | --- | --- | --- | --- | --- | --- |
|  | | Model 1 | | | | | | Model 2 | | | | | | | |
| Predictors | | B | SE | *t* | | *p* | | | B | | SE | | *t* | | *p* |
| Controls |  |  |  | |  | | |  | |  | |  | |  | |
| Age | | 0.039 | 0.040 | 0.97 | | 0.34 | | | 0.050 | | 0.040 | | 1.27 | | 0.21 |
| Gender | | 0.36 | 0.32 | 1.11 | | 0.27 | | | 0.30 | | 0.31 | | 0.95 | | 0.34 |
| Main effects |  |  |  | |  | | |  | |  | |  | |  | |
| SSS | | -0.010 | 0.10 | -0.052 | | 0.96 | | | 0.013 | | 0.10 | | 0.13 | | 0.90 |
| Resting HRV | | -0.014 | 0.30 | -0.046 | | 0.96 | | | 0.057 | | 0.30 | | 0.19 | | 0.85 |
| Interaction |  |  |  | |  | | |  | |  | |  | |  | |
| SSS x Resting HRV | |  |  |  | |  | | | **-0.65** | | 0.21 | | -3.09 | | .002 |
| Note. Estimates in bold are statistically significant. | | | |  |  | |  | | |  | |  | |  |  |


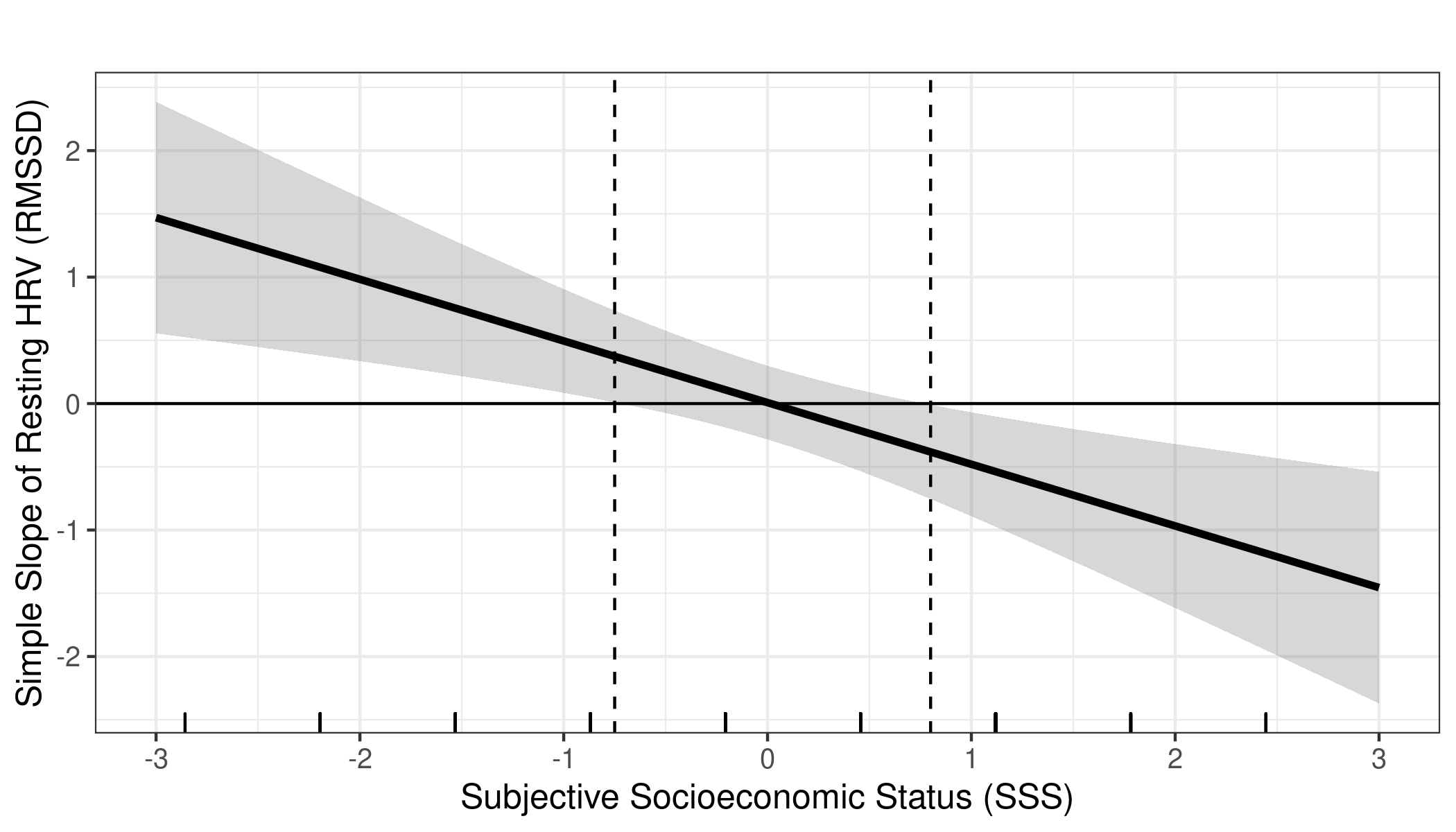


**Figure S1.** The simple slope of resting HRV on perceived pain is significant and negative when SSS is 0.8 standard deviations away from the mean or further. 21.56% of observations are within this region. The simple slope of resting HRV on perceived pain is significant and positive when SSS is -0.75 standard deviations away from the mean or further. 28.14% of observations are within this region.

For pain tolerance, similar to the analysis using raw RMSSD scores, there were no significant main effects or interaction effects of SSS and log-transformed RMSSD as HRV (including covariates) on immersion time, across all models (all *p*s > .57). We ran the same moderated path analysis (Edwards & Lambert, 2007) as reported in the main text by estimating the indirect pathways from the log-transformed RMSSD as HRV to immersion time via reported pain levels at higher SSS (mean +1 SD) and at lower SSS (mean -1 SD). Similar patterns were observed: There was a significant indirect effect of resting HRV at higher SSS, *B* = 12.78, 95% CI [1.50, 37.20], such that higher resting HRV predicted lower pain reports, *B* = -1.00, 95% CI [-2.15, -0.19], and subsequently longer immersion time (i.e., higher pain tolerance), *B* = -12.77, 95% CI [-19.53, -5.04]. At lower SSS, there was also a significant indirect effect of resting HRV but in the opposite direction, *B* = -10.02, 95% CI [=28.68, -1.21], such that higher resting HRV predicted higher pain reports, *B* = 0.94, 95% CI [0.19, 1.78], followed by shorter immersion time (i.e., lower pain tolerance), *B* = -10.62, 95% CI [-18.60, -2.27]. The overall index of moderated pathway by SSS was also significant, *B* = 22.80, 95% CI [5.18, 52.39], indicating that both pathways were different from each other.

**Analysis for perceived pain reports controlling for objective socioeconomic status (SES).** We also assessed participants’ current annual household income based on eight categories of income (*M* = 5.95. *SD* = 1.66), and their parents’ highest educational attainment based on five categories, which were averaged across both parents (Kraus & Keltner, 2009; *M* = 3.62; *SD* = 1.04), as objective measures of social class. These were additionally included as covariates to examine the unique role of SSS.

In Model 1, resting HRV, SSS, age, sex and household income did not significantly predict pain reports. There was a significant main effect of parents’ education level, such that higher parents’ educational level predicted higher pain perceived, β = 0.41, *t*(159) = 2.32, *p* = .022, 95% CI [.059, .75]. Importantly, in Model 2, the significant interaction effect between resting HRV and SSS remained, β = -.015, *t*(158) = -2.97, *p* = .004, 95% CI [-.025, -.005], *R*^2^_adjusted_ = .10. Consistent with our hypothesis, simple slopes analysis revealed that at higher SSS, higher resting HRV predicted significantly lower pain perceived, β = -.022, *t*(158) = -2.05, *p* = .042, 95% CI [-.043, -.001]. At lower SSS, an opposite pattern emerged, such that higher resting HRV predicted significantly higher pain perceived, β = .024, *t*(158) = 2.18, *p* = .031, 95% CI [.0023, .046]. The full model parameters are presented in Table S2.

| Table S2. Model Summaries of Perceived Pain with SSS and Resting HRV as Predictors (with Age, Sex, and Objective SES as Covariates). | | | | | | | | | | | | | | | |
| --- | --- | --- | --- | --- | --- | --- | --- | --- | --- | --- | --- | --- | --- | --- | --- |
|  | | Model 1 | | | | | | Model 2 | | | | | | | |
| Predictors | | B | SE | *t* | | *p* | | | B | | SE | | *t* | | *p* |
| Controls |  |  |  | |  | | |  | |  | |  | |  | |
| Age | | 0.034 | 0.040 | 0.85 | | .40 | | | 0.49 | | 0.040 | | 1.24 | | .22 |
| Gender | | 0.47 | 0.32 | 1.46 | | .15 | | | 0.38 | | 0.32 | | 1.21 | | .23 |
| Household income | | 0.012 | 0.11 | -1.80 | | .074 | | | -0.14 | | 0.12 | | -1.21 | | .23 |
| Parents education | | 0.41 | 0.18 | 2.32 | | .022 | | | 0.39 | | 0.17 | | 2.26 | | .025 |
| Main effects |  |  |  | |  | | |  | |  | |  | |  | |
| SSS | | .012 | 0.11 | 0.11 | | .92 | | | 0.024 | | 0.11 | | 0.22 | | .83 |
| Resting HRV | | 0.0001 | 0.008 | 0.031 | | .98 | | | 0.001 | | 0.008 | | 0.12 | | .91 |
| Interaction |  |  |  | |  | | |  | |  | |  | |  | |
| SSS x Resting HRV | |  |  |  | |  | | | **-0.015** | | 0.005 | | -2.97 | | .004 |
| Note. Estimates in bold are statistically significant. | | | | |  | |  | | |  | |  | |  |  |

We conducted a parallel analysis using log-transformed RMSSD as HRV. In Model 1, there were no significant effects of SSS, log-transformed RMSSD as HRV, age and sex. There was again a significant main effect of parents’ education level, such that higher parents’ educational level predicted higher pain perceived, β = 0.41, *t*(159) = 2.34, *p* = .020, 95% CI [.064, .75]. Critically, in Model 2, there was the expected significant interaction effect between HRV and SSS, β = -0.61, *t*(158) = -2.78, *p* = .006, 95% CI [-1.04, -.18], *R*^2^_adjusted_ = .099. Specifically, higher resting HRV had a non-significant tendency to predict lower pain perceived at higher SSS, β = -0.82, *t*(158) = -1.81, *p* = .072, 95% CI [-1.72, .074]—a weaker pattern than other parallel analyses. In contrast, at lower SSS, higher resting HRV predicted significantly higher pain perceived, β = 1.00, *t*(158) = 2.28, *p* = .024, 95% CI [.13, 1.86]. Despite the weaker simple slope observed at higher SSS in this model with 4 covariates, the overall patterns are still consistent. The full model parameters are presented in Table S3.

| Table S3. Model Summaries of Perceived Pain with SSS and Log-Transformed RMSSD as HRV as Predictors (with Age, Sex, and Objective SES as Covariates). | | | | | | | | | | | | |
| --- | --- | --- | --- | --- | --- | --- | --- | --- | --- | --- | --- | --- |
|  | | Model 1 | | | | Model 2 | | | | | | |
| Predictors | | B | SE | *t* | *p* | | B | SE | | *t* | | *p* |
| Controls |  |  |  |  | |  |  | |  | |  | |
| Age | | 0.035 | 0.040 | 0.88 | .38 | | 0.48 | 0.040 | | 1.21 | | .23 |
| Gender | | 0.48 | 0.32 | 1.50 | .14 | | 0.37 | 0.32 | | 1.17 | | .24 |
| Household income | | -0.22 | 0.12 | -1.81 | .072 | | -0.15 | 0.12 | | -1.26 | | .21 |
| Parents education | | 0.41 | 0.18 | 2.34 | .020 | | 0.40 | 0.17 | | 2.35 | | .020 |
| Main effects |  |  |  |  | |  |  | |  | |  | |
| SSS | | .011 | 0.11 | 0.098 | .92 | | 0.024 | 0.11 | | 0.21 | | .83 |
| Resting HRV | | .12 | 0.31 | 0.39 | .70 | | 0.075 | 0.30 | | 0.25 | | .80 |
| Interaction |  |  |  |  | |  |  | |  | |  | |
| SSS x Resting HRV | |  |  |  |  | | **-0.61** | 0.22 | | -2.78 | | .006 |
| Note. Estimates in bold are statistically significant. | | | | | | | | |  | |  |  |

**Analysis for perceived pain reports with objective socioeconomic status (SES).** For parallel analyses with household income as SES instead of SSS, there were no significant main effects or interaction effects of SSS and resting HRV (both raw and log-transformed) on immersion time, across all models (all *p*s > .31). For parallel analyses with parents’ educational level as SES instead of SSS, there were also were no significant main effects or interaction effects of SSS and resting HRV (both raw and log-transformed) on immersion time, across all models (all *p*s > .10).

**Exploratory analysis for self-reported post-test anxiety.** We explored if SSS and resting HRV also interacted to predict self-reports of anxiety after performing the cold pressor test but did not find significant parallel patterns. One issue that limits meaningful inference from this anxiety measure is that it was assessed only after the cold pressor test but not at baseline. Without a baseline measure, it is unclear if post-test anxiety levels capture increases or decreases in anxiety, as a result of the test. Therefore, the role of anxiety and this analysis were not discussed in the main paper. Nonetheless, we present the full model parameters from this analysis in Table S4, for the interested reader.

| Table S4. Model Summaries of Post-CPT anxiety with SSS and Log-Transformed RMSSD as HRV as Predictors (with Age, Sex, and Objective SES as Covariates). | | | | | | | | | | |
| --- | --- | --- | --- | --- | --- | --- | --- | --- | --- | --- |
|  | | Model 1 | | | | Model 2 | | | | |
| Predictors | | B | SE | *t* | *p* | | B | SE | *t* | *p* |
| Controls |  |  |  |  | |  |  |  |  | |
| Age | | -0.019 | 0.017 | -1.13 | .26 | | -0.015 | 0.017 | -0.88 | .38 |
| Gender | | -.020 | 0.14 | -0.15 | .88 | | -0.045 | 0.13 | -0.33 | .74 |
| Household income | | -0.032 | 0.050 | -0.65 | .52 | | -0.013 | 0.050 | -0.25 | .80 |
| Parents education | | -0.050 | 0.073 | -0.69 | 0.49 | | -0.055 | 0.072 | -0.77 | .45 |
| Main effects |  |  |  |  | |  |  |  |  | |
| SSS | | 0.030 | 0.047 | 0.64 | 0.53 | | 0.033 | 0.047 | 0.71 | 0.48 |
| Resting HRV | | -0.003 | 0.003 | -0.86 | 0.39 | | -0.003 | 0.003 | -0.81 | 0.42 |
| Interaction |  |  |  |  | |  |  |  |  | |
| SSS x Resting HRV | |  |  |  |  | | -0.004 | 0.002 | -1.94 | .054 |
